# Narrative Review of Learning Methods for Junior Doctors Around Presenting Evidence at Mental Health Review Tribunals

**DOI:** 10.1192/bjo.2024.305

**Published:** 2024-08-01

**Authors:** Kenn Cheng Keat Lee

**Affiliations:** Pennine Care NHS Foundation Trust, Bury, United Kingdom

## Abstract

**Aims:**

Introduction: During involuntary hospital commitment, patients are detained and receive treatment involuntarily without prior judicial authorisation. Instead, detentions are scrutinised after-the-fact through mental health review tribunals (MHRTs), where psychiatrists must satisfy the panel that hospital detention is the least restrictive option. Such settings are different from what doctors are typically trained to do – namely provide care to willing patients. Yet, presenting evidence at MHRTs is part of regular psychiatric practice. Thus, doctors training in psychiatry would need to learn this skill.

Objective: Review the available literature on learning methods that are effective at developing junior doctors’ capability to present evidence at MHRTs.

**Methods:**

Methodology: Seven electronic databases (Medline, Embase, PsycINFO, Web of Science, Education Source, ERIC, Westlaw UK) were searched for studies evaluating the teaching/training of junior doctors to deliver evidence at MHRTs and related settings (inquests, criminal courts), published within the last 25 years. Due to the heterogeneity in methodology, the studies were reviewed narratively.

**Results:**

2,206 articles were found, of which six met criteria (four quasi-experimental studies, two qualitative studies). All quasi-experimental studies were from the UK whilst both qualitative studies were of non-UK origin. Sample sizes were uniformly small (3–16 participants) or unclear/undocumented (2 studies). One study revolved around interprofessional learning in criminal court setting. The remainder were about MHRTs, using a mix of modalities (simulation = 2, workshop = 1, lecture with demonstration = 1, instructional document = 1). Simulation, lecture with demonstration, and workshop were effective at developing skills in oral presentation and being cross-examined. All methods were effective at developing report writing skills. However, articles mainly assessed efficacy through pre/post self-assessment of confidence without control/comparator.

Discussion: MHRT guidelines indicate hands-on learning as mainstay of how doctors develop their capabilities in MHRT. However, this is not reflected in or supported by the published evidence. Likewise, evidenced methods (e.g. simulation, workshops) are resource-intensive and may be difficult to replicate at scale. Additionally, identified articles lacked clear articulation of the pedagogy or theory underpinning the learning, though they appeared constructivist in nature.

**Conclusion:**

The literature around training junior doctors to deliver evidence at MHRT is underdeveloped. Current standard methods are not supported by evidence whilst evidence-backed methods may be difficult to implement cohort-wide. What evidence that exists is weak and based on subjective self-assessment. Further research on the topic is needed, both around standard training/learning methods and more objective methods of assessing efficacy.

